# Complete Genome Sequences of Two Newcastle Disease Virus Strains of Genotype VIII

**DOI:** 10.1128/genomeA.00180-12

**Published:** 2013-02-14

**Authors:** Yongzhong Cao, Min Gu, Xiaorong Zhang, Wenbo Liu, Xiufan Liu

**Affiliations:** Animal Infectious Disease Laboratory, College of Veterinary Medicine, Yangzhou University, Yangzhou, Jiangsu, China

## Abstract

Here, the whole genome sequences of two Newcastle disease viruses (NDV) of genotype VIII, which were isolated from west China in the 1980s, were determined and characterized phylogenetically. This is the first report with respect to the complete genomic information of genotype VIII NDV strains.

## GENOME ANNOUNCEMENT

Any outbreak of Newcastle disease (ND) requires notification to the Office International des Epizooties (OIE) (1). The pathogen of ND is Newcastle disease virus (NDV), which is a member of genus *Avulavirus* within the family *Paramyxoviridae* (2). NDV is a negative-sense single-strand nonsegmented enveloped RNA virus and has at least three genome lengths: 15,186, 15,192, and 15,198 nucleotides. The genome is composed of six genes that encode the corresponding six structural proteins from the 3′ end to the 5′ end: nucleoprotein (NP), phosphoprotein (P), matrix (M), fusion (F), hemagglutinin-neuraminidase (HN), and the RNA polymerase (L) (3). NDV strains are divided into two classes based on genetic analysis. Class I strains are isolated mainly from wild birds and are generally avirulent, whereas class II strains can be recovered from wild and domestic birds and include virulent and avirulent isolates. Both class I and class II viruses can be further categorized into 9 and 11 genotypes, respectively (4).

According to their pathogenicity in chickens, NDV strains are classified into three pathotypes, i.e., lentogenic (low virulence), mesogenic (moderate virulence), and velogenic (high virulence). Viruses of different genotypes vary in their virulence and are closely related with prevalent historical periods (4). During the past 10 years, a large number of the full genomic sequences of different genotypes of NDV, involving genotypes I to VII and IX, have been submitted to GenBank; this is quite valuable for the phylogenetic study of the evolutionary pattern of NDV. However, complete genomic information for genotypes VIII and X has not yet been retrieved.

Genotype VIII viruses were isolated from South Africa and South Asia in the 1960s, 1980s, and 1990s. In China, genotype VIII was first isolated from west China and subsequently was identified in the 1980s (5), but it has not been reported since 2000. In this research work, the genomic sequences of two Chinese isolates of genotype VIII (QH1 and QH4) were determined by a set of 10 pairs of PCR primers referring to the published NDV sequences. The genome compositions of both isolates are each 15,192 nucleotides in length, and the G+C and A+T contents are 46% and 0.54%, respectively. QH1 and QH4 are highly homogenous, with 99.9% identity in their nucleotide sequences. Phylogenetic analysis at the genome level showed that the two genotype VIII viruses had a very high correlation (90% nucleotide identity) with a genotype VI virus, IT-227/82 (GenBank accession no. AJ880277) of class II, but there was a distant relation (73%) between the two isolates (QH1 and QH4) and the DE_R49/99 strain (GenBank accession no. DQ097393) of class I. A homological comparison of the 6 structural genes indicated that the NP, P, F, HN, and L genes were individually related (92 to 95%) to the virus isolated from Malaysia (GenBank accession no. AF2240). The M gene was intimately clustered (95%) with the Trenque Lauquen virus (GenBank accession no. AF100300) isolated from Argentina.

### Nucleotide sequence accession numbers.

The genome sequences of QH1 and QH4 were deposited in GenBank under the accession no. FJ751918 and FJ751919, respectively.

## References

[1] OIE 1992 In Newcastle disease. International animal health code (mammals, birds and bees), 6th ed Office International des Epizooties, Paris, France

[2] Fauquet CM Fargette D 2005 International Committee on Taxonomy of Viruses and the 3,142 unassigned species. Virol. J. 2:64 16105179 10.1186/1743-422X-2-64 PMC1208960

[3] Czeglédi A Ujvári D Somogyi E Wehmann E Werner O Lomniczi B 2006 Third genome size category of avian paramyxovirus serotype 1 (Newcastle disease virus) and evolutionary implications. Virus Res. 120:36–48 16766077 10.1016/j.virusres.2005.11.009

[4] Miller PJ Decanini EL Afonso CL 2010 Newcastle disease: evolution of genotypes and the related diagnostic challenges. Infect. Genet. Evol. 10:26–35 19800028 10.1016/j.meegid.2009.09.012

[5] Liang R Cao DJ Li JQ Chen J Guo X Zhuang FF Duan MX 2002 Newcastle disease outbreaks in western China were caused by the genotypes VIIa and VIII. Vet. Microbiol. 87:193–203 12052330 10.1016/s0378-1135(02)00050-0

